# Immediate Maxillary Full-Arch Rehabilitation of Periodontal Patients with Terminal Dentition Using Tilted Implants and Bone Augmentation: A 5-Year Retrospective Cohort Study

**DOI:** 10.3390/jcm11102902

**Published:** 2022-05-20

**Authors:** Gil S. Slutzkey, Omer Cohen, Liat Chaushu, Arkadi Rahmanov, Eitan Mijiritsky, Ilan Beitlitum, Roni Kolerman

**Affiliations:** 1 Department of Periodontology and Oral Implantology, The Maurice and Gabriela Goldschleger School of Dental Medicine, Tel-Aviv University, Tel-Aviv 6997801, Israel; omerco2@gmail.com (O.C.); liat.natanel@gmail.com (L.C.); beilan1612@gmail.com (I.B.); 2 Department of Oral Rehabilitation, The Maurice and Gabriela Goldschleger School of Dental Medicine, Tel-Aviv University, Tel-Aviv 6997801, Israel; phenomenar18@gmail.com (A.R.); mijiritsky@bezeqint.net (E.M.); 3 Department of Otolaryngology Head and Neck Surgery and Maxillofacial Surgery, Tel-Aviv Sourasky Medical Center, Sackler School of Medicine, Tel Aviv University, Tel-Aviv 6997801, Israel

**Keywords:** immediate implants, tilted implants, periodontitis, marginal bone loss

## Abstract

Background: All-on-four protocols with tilted implants in the maxilla are used to rehabilitate the terminal dentition of the severe generalized periodontitis patients. Data on long-term biological complications are scarce. Methods: Eighty-four axial and forty-six tilted immediate implants have been placed in the extraction sockets of 23 patients according to a four–six implants protocol combined with ridge augmentation. Within 72 h, a provisional prosthesis was cemented to the implants; after 6 months, a cemented ceramic–metallic prosthesis was delivered. The patients were followed for up to 5 years. Results: The 5-year survival rate of the straight and tilted implants was 100% and 97.8, and the prosthetic one was 100%. Marginal bone loss (MBL) of the straight implants was 0.42 ± 0.67 and 0.59 ±1.01 mm on the mesial and distal sides; for the tilted, it was 0.37 ± 0.68 and 0.34 ±0.62 mm, and the differences were not statistically significant. Implant position, smoking, keratinized mucosal width, and cantilever did not affect MBL. Peri-implant mucositis involved 29.4% and 22.2% of the straight and tilted implants, respectively; peri-implantitis involved 5.8% and 4.4% of the straight and tilted implants, respectively, without statistical significance. Conclusions: This immediate loading protocol’s 5-year survival and success rates were high. No difference between the straight and tilted implants was found regarding survival, success rates, and MBL.

## 1. Introduction

The edentulous posterior maxilla is often associated with the pneumatization of the maxillary sinus and a reduced height of the alveolar ridge at sites to place implants [1]. Bone augmentation of the maxillary sinus is a standard and predictable surgical procedure in implant therapy; however, this procedure is associated with patient morbidity, possible surgical complications, high costs, and a long 6–12 month healing period [2]. Other treatment options are short implants, implant-supported fixed partial dentures with a distal cantilever, tilted implants, and implants placed in the zygoma or the tuberosity [3,4].

The use of tilted implants has gained popularity as a feasible option to treat the edentulous maxilla by means of implant-supported rehabilitation without requiring a grafting procedure [5]. With this technique, anterior implants are placed axially, and the most posterior ones are tilted to run parallel to the anterior wall of the maxillary sinus [4]. Implementing tilted implants (TIs) may offer several clinical advantages: (1) placing longer implants to increase the bone-to-implant contact and, subsequently, implant stability; (2) widening the distance between the anterior and posterior implants, and thus improving the load distribution; and (3) reducing the size of the distal cantilever or even eliminating it. The TIs require the use of angled abutments. Several in vitro studies have suggested that angled abutments increase stress on the supporting implants and the adjacent bone [6]. This strain has been claimed to increase with decreasing bone density [7]. Despite a 3.0- and 4.4-fold stress increase on the 15° and 25° angled abutments, stress on the bone is tolerated and remains within the physiological limits compared with the straight abutments [7]. Most patients with a failing dentition express the desire to undergo a treatment protocol that involves immediate implant placement and restoration because discomfort, treatment time, and cost are reduced. Moreover, it eliminates the need for a transitional removable prosthesis and preserves the height and width of the residual alveolar ridge [8,9].

This treatment modality, of a fixed screw-retained restoration supported by implants placed immediately after tooth extraction in a single surgical procedure using axial and tilted implants, has been well documented; it is known as the all-on-four/all-on-six protocol [10,11,12]. The main disadvantages of this treatment protocol are the difficulty of placing implants in extraction sockets with sufficient insertion torque, the unpredictable resorption extent of the buccal bone during the first six months of healing, and the inability to control the width of the keratinized tissue. Additionally, a monthly prosthetic evaluation and a complex hygienic maintenance program are needed due to the bolted rehabilitation pattern with pink cervical porcelain in the format described by Malo et al. [12]. The need to insert the implants at sites that offer the best mechanical anchoring conditions turns the surgery into that which is not prosthetically driven.

Nowadays, the challenge is not to prove feasibility and success but rather to develop cost-effective and straightforward protocols. The all-on-four concept has been documented by several studies and clinical reports [13,14,15]; however, in the year 2015, when the present study was started, the data were mainly limited to survival rates, implant failure, and technical complications. Little emphasis was placed on biological complications like peri-implantitis and peri-implant mucositis, which are currently considered frequent events [13]. More recent review papers have been focusing on biological complications [14] and patient satisfaction with the treatment [15]; for example, in a 5-year follow-up study of immediately loaded full-arch rehabilitations, tilted implants were shown to accumulate more plaque than axial implants [16]. In the periodontally compromised patient, MBL was higher by 0.60 mm when compared to the healthy periodontal patient, and the risk ratio of implant failure was 1.78 [17]. Another review reported a similar 0.61 mm MBL difference between the healthy periodontal patients and the affected ones [18]. The latest meta-analysis claimed that aggressive periodontitis and chronic periodontitis patients present a 4.4 and 1.6 higher risk of implant loss, respectively, than periodontally healthy ones [19]. Nonetheless, the long-term documentation of a surgical and prosthetic protocol involving the immediate placement, hard tissue augmentation, and immediate loading with a cement-retained provisional reinforced acrylic bridge relying on four–six implants in the maxilla to rehabilitate the periodontal patient with a terminal maxillary dentition is scarce [14,20]. It has been suggested that the way to reach a balanced and stable esthetic outcome of the soft and hard tissues in the aesthetic area is to use an osteoconductive bone substitute, such as a freeze-dried bone allograft, and a resorbable membrane to prevent an extensive bone remodeling from taking place [8,9]. This technique was previously described for single tooth replacement and multiple extractions and implantation sites; it enabled improving the labial/buccal contours without interfering with the natural healing capability of the alveolar bone following extraction [8,9]. The rationale behind it is that slowly resorbing or nonresorbing bone substitute particles are incorporated into the soft tissues, thereby preventing recession and enhancing the soft tissue appearance of the edentulous ridge [8,9].

Subsequently, the aim of the present 5-year retrospective study was to:

(1) Evaluate the survival and success rates of this treatment protocol in stage 4 grade C generalized periodontitis patients [21];
(2) Compare the MBL of tilted and axial immediately loaded implants placed concomitantly with bone augmentation;
(3) Assess the impact on the MBL of other covariates, such as smoking, bruxism, cantilever, implant location, and the presence of keratinized mucosa.

We hypothesized that high survival and success rates for both the axial and tilted implants and similar marginal bone losses would be achieved.

## 2. Materials and Methods

### 2.1. Patients Selection

This survey covers all consecutive patients treated with the all-on-4–6 protocol by one periodontist (RK) over 7 years. All the participants meet the inclusion criteria described below. The treatment consisted of extracting the existing maxillary teeth and placing 4–6 immediately loaded implants in combination with bone augmentation. All patients signed an informed consent form to allow the retrospective use of their clinical data. The study was approved by the ethics committee of Tel Aviv University (no. 0000030-1).

#### 2.1.1. Inclusion Criteria

1. A diagnosis of generalized stage 4 grade C by a periodontist according to the new classification of periodontal diseases from 2018, based on clinical examination and radiographic evaluation [21];
2. Need for the extraction of all upper teeth, primarily due to periodontitis. Other reasons included extensive carious lesions and root/tooth fractures;
3. Mandibular teeth or implants sustaining a fixed partial or complete arch restoration or consisting of natural dentition with a good or fair prognosis;
4. Age of ≥18 years;
5. Full mouth plaque score <25% [22];
6. Stable periodontal disease of the mandibular teeth;
7. ≥5 mm bone apical or palatal to the alveolar socket;
8. Primary stability with an insertion torque of ≥30 Newton centimeter (Ncm);
9. Full or partial integrity of the socket walls after extraction;
10. Cone-beam tomographic (CBCT) examination was obtained before surgery.

#### 2.1.2. Exclusion Criteria

1. Poorly controlled diabetes mellitus with uninterrupted hemoglobin A1c > 8.0% for ≥1 year despite standard care;
2. Current or past metabolic bone disease;
3. Medical treatment with bisphosphonates (both oral and IV);
4. A history of radiotherapy or chemotherapy to the head and neck region;
5. Pregnancy or lactation;
6. Parafunctional habits (e.g., bruxism or clenching);
7. Lack of compliance.

The opposing mandibles of 10 cases (44%) were full-arch implant-supported restorations, 4 (17%) had natural teeth or teeth-supported restorations, and 9 (39%) had combined implant-supported restorations.

### 2.2. Surgical Treatment

The presurgical evaluation included demographic and medical data, smoking, full mouth periapical radiographs, periodontal chart, occlusal analysis, and periodontal diagnosis.

Before treatment of the maxilla, the natural mandibular teeth underwent complete periodontal treatment, including preliminary cause-related therapy and additional periodontal surgery if needed.

**Figure 1:**
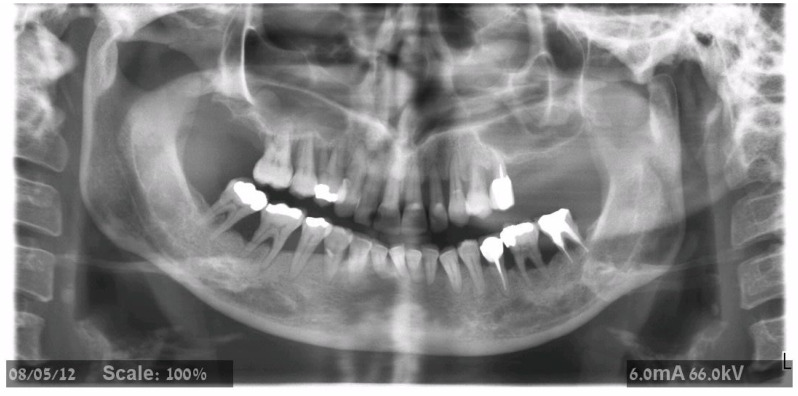
Panoramic X-ray showed severe bone loss of the upper dentition. Bone height in the posterior maxilla did not allow implant insertion without a sinus augmentation procedure.

**Figure 2:**
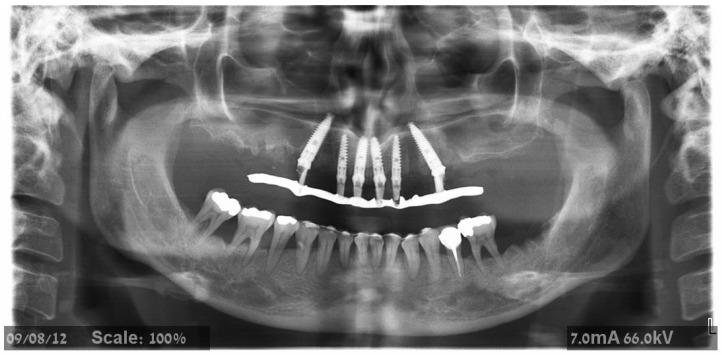
Panoramic X-ray on the day of provisional prosthesis delivery. Two posterior tilted implants were placed on each side to avoid sinus augmentation.

**Figure 3:**
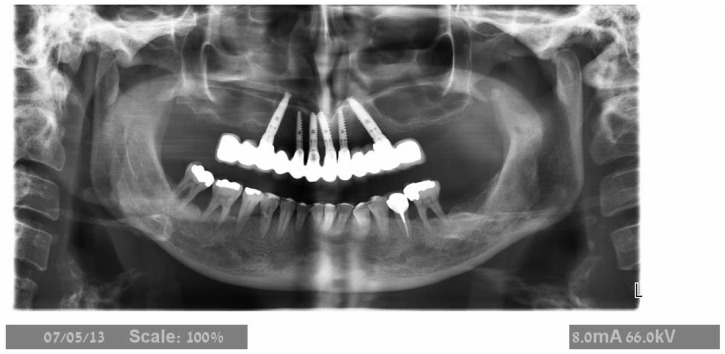
Panoramic X-ray after implant placement with the final prosthesis. The prosthesis was designed with two distal cantilevers on the right side and one on the left.

The maxillary teeth to be extracted were gross-scaled, and the patients were instructed about proper plaque control. A recent CBCT was available before surgery in all cases. Patients were asked to begin 0.2% CHX mouth wash several days before surgery; one hour before surgery, the patients were given premedication with 875 mg amoxicillin–clavulanate potassium (Augmentin, Smith Kline, Brentford, UK) or 600 mg clindamycin HCl (Dalacin-C, Pfizer NV/SA, Puurs, Belgium) and 8 mg dexamethasone (Aspen, Dublin, Ireland). Before the procedure, a one-minute rinsing with a 0.2% chlorhexidine solution (Tarodent mouthwash, Taro Pharmaceutical Industries, Haifa, Israel) was completed. Patients continued taking antibiotics for one week after surgery (Augmentin 875 mg × 2 per day or Dalacin 150 mg × 4 per day). The 4 mg dexamethasone treatment was carried out over two successive days. Starting the day after treatment, the patients were instructed to rinse with 0.2% chlorhexidine twice a day for two weeks. A panoramic examination was performed (Figure 1, Figure 2 and Figure 3) at 3-time points: before treatment, immediately after implant placement, and after bridge installation.

**Figure 4:**
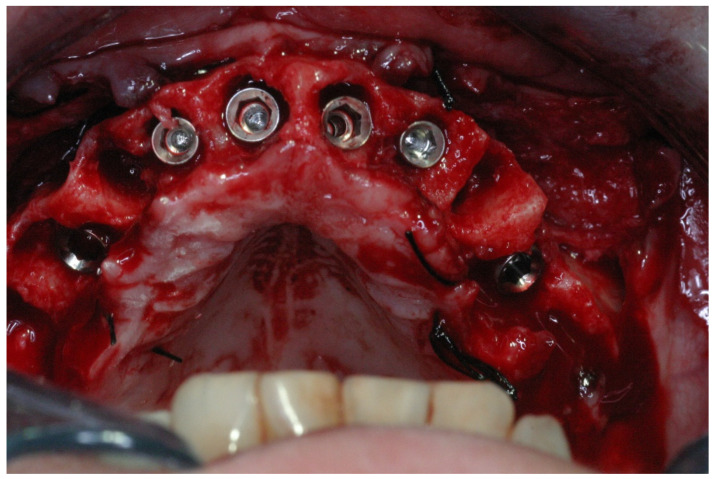
Implants were placed 2–3 mm palatal to the buccal wall of the extraction socket, and the implant’s shoulder was placed 0–2 mm apical to the palatal bone.

One surgeon (RK) completed all surgeries. After local anesthesia, intrasulcular incisions and elevation of a full-thickness flap were carried out. Subsequently, extractions were performed, and the granulation tissue was removed. All implants were placed in socket sites without using a surgical guide. Implant osteotomy was prepared by engaging the palatal and apical walls of the socket; no attempt was made to level the bone. Depending on the bone quality, the final drill was at least 1 mm smaller in diameter than the implant width to attain the desired primary stability. Implants, Lance or Seven (MIS) with a sandblasted and acid-etched surface (MIS Implants Technologies, Bar-Lev industrial zone, Israel), were inserted with a torque-controlled ratchet; primary stability was considered sufficient when the insertion torque was ≥ 30 Ncm. Implants were placed 2–3 mm palatal to the buccal wall of the extraction socket and at the level of the palatal bone or slightly apically (Figure 4).

**Figure 5:**
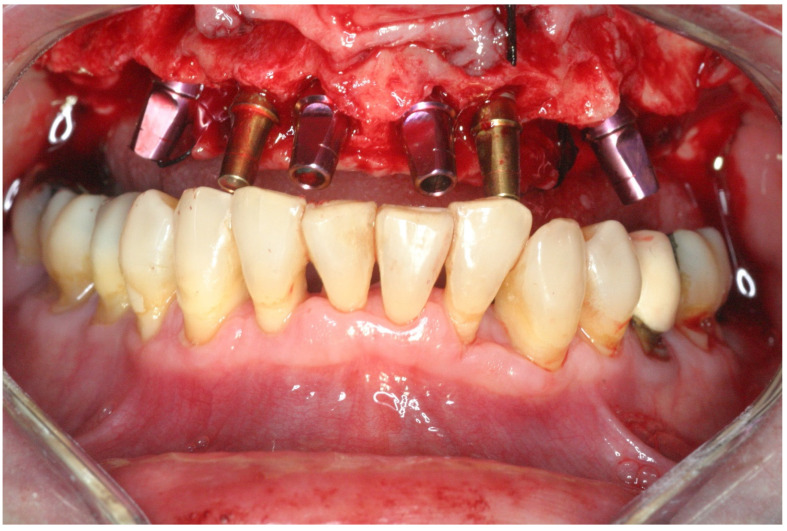
Color-coded transfers adapted after implant placement.

**Figure 6:**
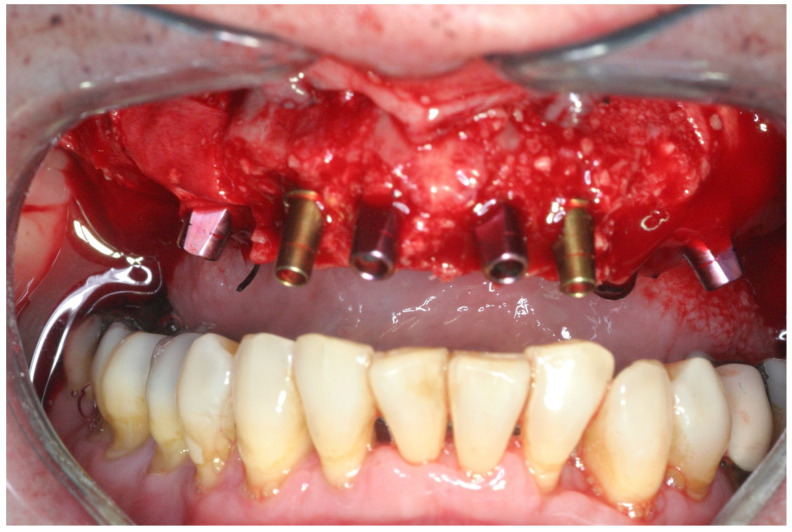
Particulate bone allograft (FDBA) was placed in the residual gap and above the buccal bone to compensate for the anticipated bone loss. A resorbable collagen membrane was adapted to the augmented buccal bone and regenerated socket. Frontal view.

**Figure 7:**
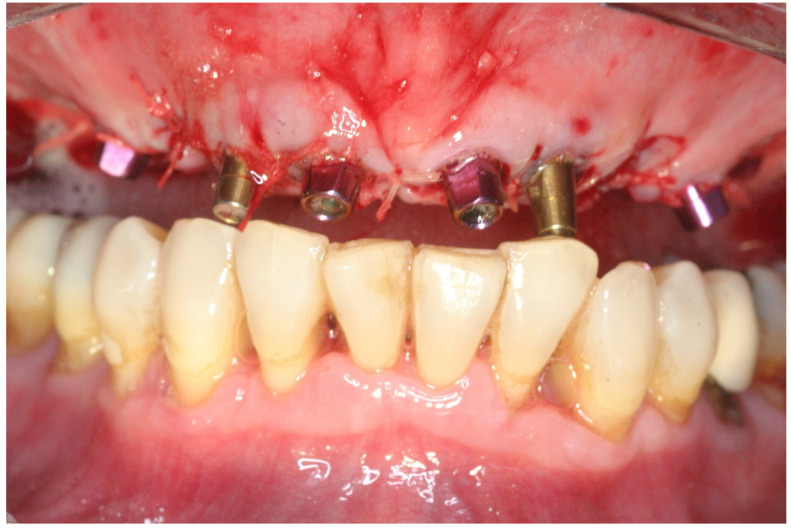
The buccal flap was released and sutured using 4/0 resorbable sutures.

Implant transfer adaptation was made immediately after all implants were placed (Figure 5); control radiographs were conducted to confirm full seating. Afterward, the sockets and the ridge were augmented with a particulate freeze-dried bone allograft (FDBA) (0.25–1 mm, Life-Net, Virginia, FL, USA), which filled the residual gaps around the implants and was added in excess above the buccal bone in sites with less than 2 mm of thickness. A collagen membrane (Bio-Gide Geistlisch Pharma AG, Wolhusen, Switzerland) covered the augmented areas (Figure 6). Tension-free suturing was realized after periosteal releasing incisions of the buccal flap. The flaps were adapted using the horizontal mattress and a simple interrupted suture technique (4/0 Vicryl rapid, Ethicon, Johnson & Johnson, New Brunswick, NJ, USA) (Figure 7). After suturing, plastic impression cap lock devices were inserted, and an impression was taken using the putty-wash one-step technique (Express, 3M ESPE Dental Products, St. Paul, MN, USA) with a closed metal stock impression tray. Finally, interarch relations were recorded.

Most sutures were gone on the 14th day follow-up visit, and the remaining ones were easily removed.

### 2.3. Prosthetic Protocol

One to three days after surgery, abutments were torqued at 15–20 Ncm according to implant diameter. Full seating was verified radiographically, and a prefabricated metal-reinforced temporary acrylic bridge was then delivered.

**Figure 8:**
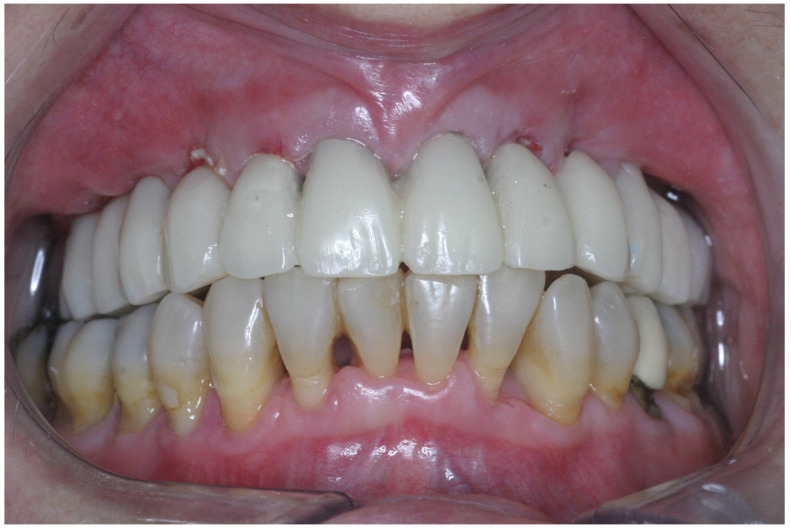
Frontal view of the provisional prosthesis.

**Figure 9:**
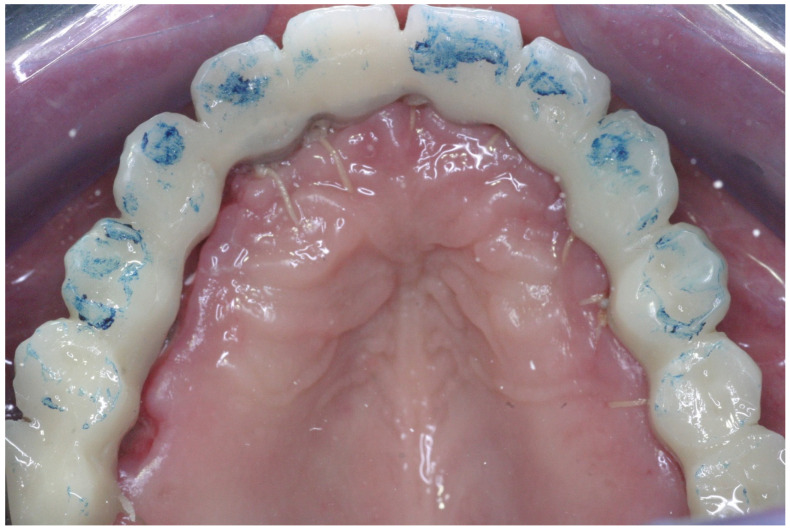
Occlusal view of the provisional prosthesis. Occlusal adjustment demonstrates maximum occlusal contact in centric relation, multipoint contacts during lateral and protrusive movements, and no occlusal contact on the distal cantilever area.

The finish line of the temporary crowns was no more than 1 mm subgingival to facilitate the removal of the residual cement and facilitate plaque control. The temporary and final bridge design complied with the prosthetic principles aimed at reducing the overload, limiting the cantilever length (≤15 mm), preparing flat cusp inclines, and organizing multiple contact points centered on the crown of various teeth to increase the distribution of force, and avoiding interferences [23]. Depending on the emerging positions of the most distal implants, the provisional prostheses comprised 10–12 units; occlusal adjustments were made according to the following four principles: (1) maximum contacts in centric relation, (2) multipoint contacts during lateral and protrusive movements, (3) canine guidance; and (4) a distal cantilever without contact in any position (Figure 8 and Figure 9).

**Figure 10:**
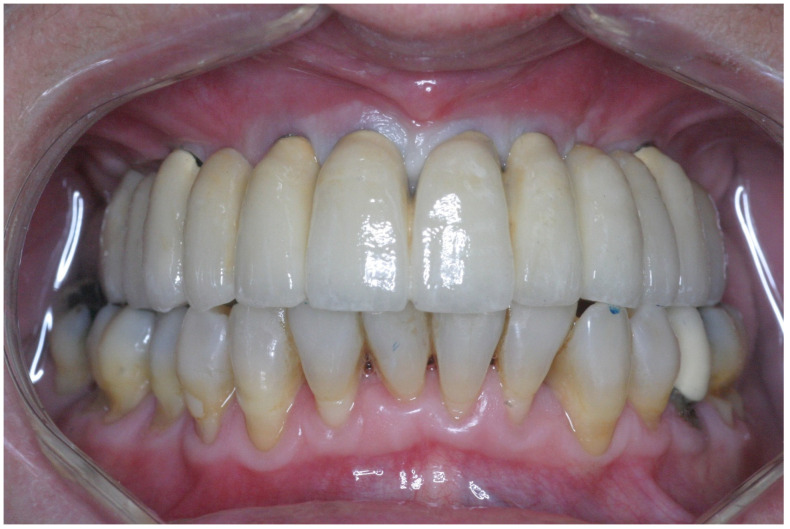
Frontal view of the final porcelain fused to a metal prosthesis cemented with temporary cement.

The provisional restoration was removed after six months of healing; the same impression protocol was used for the final restoration as for the provisional one. This restorative phase included using a master model with a silicon image of the marginal gingiva and new abutments for which the crown margins were located <2 mm subgingivally to allow an esthetic emergence profile and enable the removal of cement excess. The abutments were connected during the next appointment, and porcelain fused to a metal bridge with embrasure spaces that allowed the use of interproximal brushes was tried. Then, a ratchet was used to tighten the abutments to 15–35 Ncm (depending on implant diameter). Cementation of the permanent porcelain fused to a semiprecious metal bridge (Figure 10) was achieved with temporary cement (Temp-Bond, Kerr Corporation, Ann Arbor, MI, USA).

#### Clinical Follow-Up Examination

Supervised dental hygienists performed periodical 3–6 months supporting periodontal treatment (SPT) appointments. They included recording the plaque index, probing depth, bleeding, personal oral hygiene instructions, and scaling and root planning, if needed. The senior author (RK) performed the full periodontal evaluation once a year. Since the prosthetic design enabled full access to the implant circumference, maintenance treatment was performed without removing the prosthesis.

### 2.4. Outcome Measurements

Outcome measurements included:

Implant success rates according to Albrektsson et al. were [24].

- No pain;
- Bone loss during the 1st year <1.5 mm;
- Annual bone loss <0.2 mm thereafter;
- No peri-implant radiolucency;
- No implant mobility;
- No signs of infection.

#### 2.4.1. Implant-Related Complications

- Peri-implant mucositis was defined as an inflammatory lesion of the mucosa surrounding the implant without loss of supporting bone. The clinical signs of inflammation were bleeding on probing (BOP), while additional symptoms may include erythema, swelling, and suppuration [25].
- Peri-implantitis was defined as clinical signs of inflammation, including redness, edema, mucosal enlargement, BOP with or without suppuration along with increased PD (≥6 mm), and progressive radiographic bone loss ≥3 mm [26].

#### 2.4.2. Prosthetic Complications

1. Mechanical complications related to the loosening of abutments or decementation of the temporary or final bridge;
2. Esthetic complications evaluated by the patient in terms of lip support and appearance of the artificial teeth;
3. Functional complications, cheek and lip biting, and phonetic complaints.

#### 2.4.3. Periodontal Parameters at the Last Recall Visit

- Plaque index (PI)—Using a disclosing solution, the plaque was measured at four sites per implant, and the percentage of visible plaque was calculated [22].
- Bleeding index (BI)—A yes/no reading of bleeding within 10 s after probing at four sites for each implant was done. The bleeding index was calculated per patient (total number of bleeding sites divided by the number of implants ×4).
- PD—The probing depth was measured to the nearest mm at four sites at the follow-up examinations. The mean implant probing was calculated and used for statistical analysis.
- Keratinized mucosal width (KMW) was measured with a probe to the nearest mm; PD, BI, and PI were measured at the mesial, distal, buccal, and palatal sides.

#### 2.4.4. Radiographic Measurements

**Figure 11:**
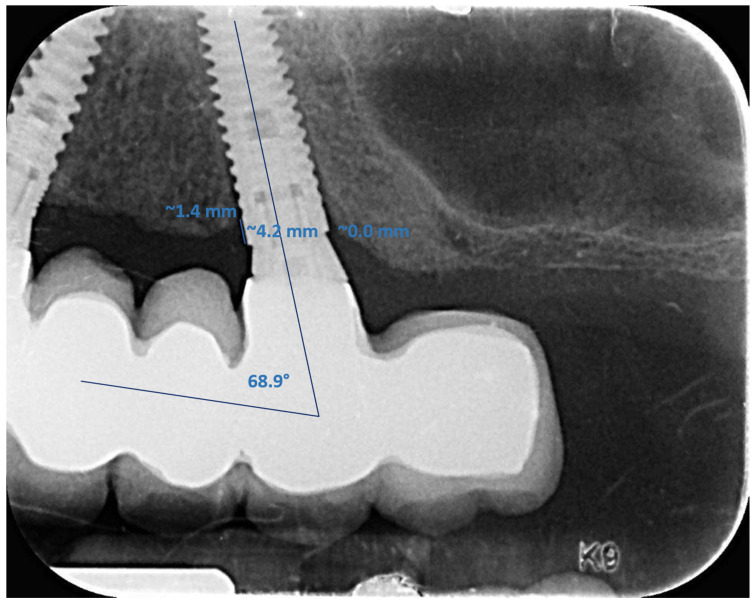
Measurement of the marginal bone level and the angle of the tilted implant with dedicated software. After calibration, the implant shoulder was used as a reference level (RL), and the distance from the RL to the first bone-to-implant contact was measured. The angle of the tilted implants was measured by tracing lines through the occlusal plane and parallel to the long axis of the implants.

Postoperative periapical radiographic examinations were performed at bridge installation and the annual follow-up examinations. Standardized radiographs, with the film, kept parallel (Kodak Ektaspeed plus, Eastman Kodak Co., Rochester, NY, USA) and the X-ray beam perpendicular to the implant were taken using plastic film holders (Dentsply-Rinn Corporation, Elgin, IL, USA). The bone level associated with the implants was evaluated using computerized digital radiography (Schick Technologies, New York, NY, USA) (Figure 11). Radiographic evaluations were performed by an independent examiner (AR). Evaluations were performed by measuring the distance between the marginal bone apical to the implant shoulder and the implant shoulder, which served as a reference level (RL). The distance from the RL to the first bone-to-implant contact was measured on the mesial and distal sides of the implant.

Radiographic distortion was calculated by dividing the radiographic implant length by the actual implant length. Bone loss was measured at 1, 3, and 5 years after prosthetic delivery. The grafting material induced a heavy masking effect on the peri-implant bone levels on the radiographs taken immediately after surgery. Subsequently, it was necessary to use a later time reference to compare the MBL of the axial and tilted implant, and the 1-year recall after bridge installation was decided to serve as the initial reference milestone. The difference (ΔH) between the final (5 years) and the initial measurements (1 year) was calculated. Since marginal bone loss is time-dependent, both the annual bone loss and the raw (total) MBL at the last follow-up were determined.

When the marginal bone was coronal to the implant shoulder, it was considered a zero-bone loss; only changes apical to the implant shoulder were measured. Implant angulation was measured by tracing two lines, one through the occlusal plane and the other parallel to the long axis of the implant (Figure 11). Angulation between each implant and the occlusal plane was calculated by subtracting the intersection angle from 90 degrees. The average angulation of the axial and tilted implants and the average annual mesial and distal bone loss for the axial (A) and tilted (T) implants were calculated.

### 2.5. Statistical Analysis

The primary outcome variables were the survival and success rates of the axial and tilted implants and the MBL between the 1- and 5-year recall. The ANOVA test with repeated measures was used in a mixed statistical analysis model, taking into account that each patient had several implants. The model compared the MBL rate between implants displaying an angulation of <15° and ≥15°, implants supporting or not supporting a cantilever, sites with and without KTW, smokers and nonsmokers, and implant location. The SPSS version 25.0 (IBM, Armonk, NY, USA) was used for the statistical analysis.

## 3. Results

### 3.1. Demographic Data

Twenty-three consecutive patients, eleven men and twelve women, met the inclusion criteria. The mean age was 65.34 ± 10.46 years at implant placement (range 46–90). Eight patients were smokers (two of them ≥ 10 cigarettes/day) (Table 1).

Eighty-four straight implants and forty-six tilted implants were placed in the postextraction sockets of the maxilla. The first molar was the most common site for the tilted implants (52%). The distribution of implants according to type, diameter, and length is shown in Table 2. Of the 130 implants (MIS Dental Implant Technologies, Bar-Lev Industrial Park, Israel), 112 were Lance (86.2%) and 18 were Seven (13.8%). The most used implant length was 16 mm (*n* = 93, 71.5%) for both the axial and tilted implants; the most frequent implant diameter was Ø 3.75 mm (67/130, 51.5%). The schematic outline of the study is described in Table 3.

### 3.2. Implant Survival and Success Rate

No implant was lost during the first and third years, and the survival rate was 100%. One tilted implant failed afterward, and the 5-year survival rate of the straight and tilted implants was 100% and 97.8% (*p* > 0.05), respectively; our hypothesis was confirmed. The prosthetic survival rate was 100%. The total (straight + tilted) 5-year success rate, according to Albrektsson et al. [24], was 94.6%.

### 3.3. Complications

#### Biological Complications

Subnasal and suborbital hematomas occurred in 10 patients (43.4%) during the first week postsurgery. The mechanical, functional, and biological complications data are summarized in Table 4.

Peri-implantitis involving seven implants (two of them tilted) was diagnosed in three patients, of whom one was a nonsmoking 64-year-old woman that had four involved implants (one tilted implant failed). Those implants presented redness, edema, mucosal enlargement, deep periodontal pockets with bleeding upon probing, and progressive bone loss. The implants were treated with nonsurgical therapy and maintained function (Table 4). The failed implant was extracted, and the abutment hole was closed using composite resin. The bridge was recemented on five implants.

In the case of decementation, the temporary cement was replaced with Cem-Implant (B.J. M laboratories, Ann Arbor, MI, USA).

Two female patients complained about the height of the crowns of their prosthesis; over time, they became accustomed to the change, and no intervention was needed (Table 4). Two other female patients experienced lip or cheek biting; this was solved by minimal selective grinding of the lower relevant teeth to increase the overjet dimension (Table 4).

### 3.4. Marginal Bone Loss

The 5-year MBL of the mesial and distal sides of the axial implants was 0.42 ± 0.67 mm and 0.59 ±1.01, respectively; for the tilted implants, it was 0.37 ± 0.68 mm and 0.34 ±0.62, respectively. The differences between the straight and tilted implants were not statistically significant; *p* was 0.67 and 0.09 for the mesial and distal sides, respectively, and our hypothesis was confirmed. For the straight implants (*n* = 84), the mean annual MBL rate was 0.08 ± 0.13 mm for the mesial side and 0.12 ± 0.20 mm for the distal one (Table 5); for the tilted implants (*n* = 45), it was 0.07 ± 0.14 mm and 0.07 ± 0.12 mm for the mesial and distal sides, respectively (Table 5). One tilted implant did not survive the 5-year follow-up examination due to severe peri-implantitis. The difference in the MBL annual rates between the axial and tilted implants was not statistically different (*p* = 0.629 for the mesial side and *p* = 0.083 for the distal one).

A comparison of the mean MBL rate (mm) as a function of different covariates is presented in Table 5; implant position, implants with or without a cantilever, and smoking status did not affect the MBL rate.

### 3.5. Periodontal Parameters

The 5-year mean PD was 4.2 ± 1.25 mm (range 2–8), the mean BI was 27.3 ± 12.04 (range 5–45), and the mean PI was 22 ± 10.5 (range 5–38).

## 4. Discussion

In the present study, the 5-year total survival rate of immediate implants supporting a maxillary prosthesis relying on four or six immediately loaded axial and tilted implants in periodontally compromised patients was 99.2%. This finding is like or slightly superior to the previous reports on this type of restoration [27,28,29].

Most studies dealt with screw-retained restorations [30]; in contrast, the restorations of this study were cemented. The advantages of a cemented restoration include the compensation of improperly inclined implants, easier achievement of passive fit, and an intact occlusal table without a screw-access hole resulting in better control of the occlusion [30]. On the other hand, cement excess is related to more biological complications [31]. This was the reason for placing the crown margins <2 mm subgingivally, enabling control and removal of the cement excess. Previous data showed that undetected cement excess increased 3–4-fold when the restoration margin was 2–3 mm subgingivally compared to 1 mm [31].

Our findings are in line with previous studies showing no correlation between the implant location/angulation and MBL or survival rates [27,32,33].

Most authors reported an MBL ranging from 1.30 to 1.72 mm at the 3- to 5-year follow-up for the axial and tilted implants supporting a screw-retained fixed complete denture prosthesis (FCDP) [11,12,28,29]. The 5-year MBL of the present implants placed in patients diagnosed with advanced periodontitis, extensive bone loss, and terminal maxillary dentition was 0.41 mm and 0.50 on the mesial and distal sides, respectively, with no difference between the axial and tilted implants. The current periodontal and hygiene-oriented bridge construct may have contributed to the superior bone results; it is probably due to the morphology of the final restorations that enabled reasonable plaque control and full access to the whole circumference of the abutment/crown interface with electrical/manual brushes. Noteworthy, all patients of this cohort adhered to an individualized SPT, which correlated with a lower incidence of implant and bone loss [34].

Peri-implantitis involved only 13% of the patients and 5.4% of the implants compared to 22% of the patients in another study that used a computer-guided surgical protocol (Nobel Guide) and immediately loaded implants with a screwed-retained prosthesis followed for 5 years [11].

Our technical complications rate was lower than that presented by Lopes et al., who reported mechanical complications in 81.9% of patients, of which fractured prosthesis and abutment loosening contributed to 59.4% and 60.3%, respectively [11]. In line with the superior results of the current study using cemented restorations, a systematic review by Weber et al. [35] in partially edentulous patients concluded that the success rates of screw-retained and cement-retained implant-supported FDPs after a follow-up period of 6 years were 93.2% for the cemented and 83.4% for the screw-retained restorations.

The low PD (4.20 ± 1.25 mm) measured during the last follow-up examination indicated the presence of a healthy peri-implant soft tissue; this feature is most likely related to the high level of maintenance and the characteristics of the prostheses that facilitated the maintenance of the implants, as already reported in our previous studies [9,36].

Alveolar ridge volume has been found to improve with a range of different bone substitutes [37]; preclinical data [38,39] have demonstrated that the use of allografts covered with a collagen membrane may limit vertical bone loss. Limited resorbability of the grafting material can also be advantageous, as it minimizes resorption of the buccal bone [40]. In the present study, we used an FDBA inside and outside the sockets covered by a resorbable collagen membrane. This fact may have contributed to the minimal MBL and high success rate of these implants.

The presence of a distal cantilever did not increase the MBL rate of the tilted implants. This is in line with a recent meta-analysis by Freitas et al. that did not find a correlation between the presence of a cantilever and MBL in fixed partial [41] or full-arch implant-supported restorations [42].

### Limitations of This Study Are

1. Only patients who had been followed for 5 years were included, and the number of patients was therefore limited;
2. Longer follow-ups are warranted, since biological complications are time-dependent;
3. The present study was retrospective and covered all consecutive patients treated with the all-on-four–six protocols by a single periodontist; thus, no power was calculated. Nevertheless, a recent systematic review included all studies with a minimum of 20 patients [15].

The strengths of the study were:

1. A 25-year experienced periodontist performed the surgeries;
2. The treatment protocol was performed according to the state-of-the-art knowledge, as mandibular teeth were without active periodontal disease;
3. All implants were placed in the maxilla, using the same implant system, bone substitutes, and resorbable membrane in highly compliant patients.

## 5. Conclusions

Within the limitations of this study, we can conclude that our results indicate that if the prerequisites for immediate loading such as high primary stability of ≥30 Ncm and splinting of the implants via a provisional prosthesis and the use of bone level implants with a sandblasted and acid-etched surface are fulfilled:

1. Full-arch fixed restorations supported by a combination of axial and tilted implants can be a viable treatment option to rehabilitate the terminal dentition of the severe generalized periodontitis patients;

2. Neither the implant survival rate nor the peri-implant marginal bone loss seems to be affected by the inclination of the implants with respect to the occlusal plane;

3. The treatment predictability and clinical outcomes of axial and tilted implants seem comparable.

Further prospective controlled studies using the present prosthetic protocol with more extended follow-up periods are warranted to evaluate this type of treatment in patients with periodontitis.

## Figures and Tables

**Table 1:** Overview of each patient’s implant data, including: 1. Smoking; 2. Number of implants; 3. Location (FDI system); 4. Number of axial and tilted implants; 5. Cantilever; 6. Implant failure.

Patient No.	Smoking	Position of Axial Implants	Position of Tilted Implants	Cantilever	Failure
1	no	14,12,21,23	15,25	16,26	none
2	no	13,11,21,23	16,26	17,27	none
3	yes	12,11,21,22	15,25	16,26	none
4	yes	14,11,21,24	16,25	26	none
5	yes	14,12,21,23	15,25	16,26	none
6	no	14,12,22,24	16,26	17	none
7	no	14,12,22,24	16,26	17,27	26, 5 years
8	no	14,12,22,24	16,26	none	none
9	no	14,12,22,24	16,26	17,27	none
10	no	13,11,21,23	15,25	16,26	none
11	no	14,12,22,24	16,26	17,27	none
12	yes	14,12,22,24	16,26	none	none
13	yes	14,12,22,24	16,26	none	none
14	yes	14,12,22,24	16,26	17	none
15	no	14,11,22,24	16,26	17,27	none
16	no	12,22	14,24	15,16,25,26	none
17	no	12,22	14,24	15,16,25,26	none
18	yes	12,22	15,25	16,17,26	none
19	no	14,12,24	16,26	17,27	none
20	no	13,12,22,23	15,24	16,25,26	none
21	no	12,22,24	14,26	15,16	none
22	yes	14,12,22,24	15,25	16,26	none
23	no	13,11,21,23	15,25	16,26	none

**Table 2:** Distribution of implants according to diameter and length, in mm.

	Implant Diameter	Implant Length			
Implant System	(mm)	11.5	13	16	Total
MIS Lance	3.3		3	6	9
	3.75	3	15	46	64
	4.2	1	9	24	34
	5		3	2	5
MIS Seven	3.3		3	10	13
	3.75			3	3
	4.2			2	2
Total		4	33	93	130

**Table 3:** Schematic outline of the study.

Treatment Time	Preliminary Visits	Baseline Visit	24–72 h	6.5–7 Months	Follow-Up 1,3,5 Years
Screen	x				
Admission criteria	x				
Informed consent	x				
Demographics	x				
Medical history	x				
Periapical parallel X-ray	x	x	x	x	x
Cone-beam tomography	x				
Periodontal examination	x				x
Periodontal treatment—mandible	x			x	x
Surgery: extractions, implants, bone augmentation, impressions		x			
Reinforced acrylic temporary bridge delivery			x		
Final porcelain fused to metal bridge delivery				x	
Supportive periodontal treatment		x		x	x
Adverse/complications events		x	x	x	x

**Table 4:** Distribution of complications.

**Mechanical complications**	**Number of patients**	**Occurrence rate**
Provisional bridge loosening	8	34.7%
Abutment screw loosening	6	26.0%
Final bridge decementation	9	39.1%
**Functional complications**	**Number of patients**	**Occurrence rate**
Phonetic problem	5	21.7%
Crown height complaint	2	8.7%
Lip or cheek biting	2	8.7%
**Biological complications**	**Number of implants**	**Occurrence rate**
**Peri-implantitis**		
Total	7	5.4%
Straight	5	5.8%
Tilted	2	4.4%
Subject base	3	13%
**Peri-implant mucositis**	**Number of implants**	**Occurrence rate**
Straight	25	29.4%
Tilted	10	22.2%
Subject base	10	43.4%
**Failure**		
Tilted	1	0.8%

**Table 5:** Comparison of mean marginal bone loss rate (mm/year) by different parameters.

		Mesial Aspect		Distal Aspect	
Parameter (No. of Implants)		Mean	±SD	Mean	±SD
**Implant angle**	<15° (85)	0.08	0.13	0.12	0.20
	≥15° (44)	0.07	0.14	0.07	0.12
	*p*	0.63		0.08	
**Implant position**	1 (13)	0.07	0.13	0.20	0.30
	2 (34)	0.07	0.11	0.07	0.12
	3 (10)	0.15	0.18	0.17	0.21
	4 (33)	0.09	0.14	0.12	0.20
	5 (16)	0.08	0.15	0.11	0.17
	6 (23)	0.07	0.14	0.04	0.08
	*p*	0.64		0.21	
**Smoking**	no (85)	0.11	0.15	0.14	0.21
	yes (46)	0.04	0.08	0.04	0.09
	*p*	0.08		0.10	
**KTW**	<2 mm (15)	0.14	0.17	0.17	0.22
	≥2 mm (114)	0.07	0.13	0.09	0.17
	*p*	0.03		0.10	
**Cantilever**	without (6)	0.01	0.03	0.00	0.00
	with one pontic (28)	0.10	0.16	0.09	0.15
	with two pontics (7)	0.07	0.13	0.07	0.12
	*p*	0.50		0.17	

## Data Availability

Data supporting reported results can be found in the tables.

## Abbreviations

MBL	Marginal bone loss
TI	Tilted implant
KMW	Keratinized mucosal width
CBCT	Cone-beam computed tomography
FDBA	Freeze-dried bone allograft
SPT	Supporting periodontal treatment
RL	Reference level
FCDP	Fixed complete denture prosthesis
